# Deep Learning with LPC and Wavelet Algorithms for Driving Fault Diagnosis

**DOI:** 10.3390/s22187072

**Published:** 2022-09-19

**Authors:** Cihun-Siyong Alex Gong, Chih-Hui Simon Su, Yuan-En Liu, De-Yu Guu, Yu-Hua Chen

**Affiliations:** 1 Department of Electrical Engineering, School of Electrical and Computer Engineering, College of Engineering, Chang Gung University, Taoyuan 33302, Taiwan; 2 Portable Energy System Group, Green Technology Research Center, College of Engineering, Chang Gung University, Taoyuan 33302, Taiwan; 3 Department of Neurosurgery, Chang Gung Memorial Hospital, Linkou, Taoyuan 33302, Taiwan

**Keywords:** vehicle early fault diagnosis, machine learning (ML), linear predictive coefficient (LPC), wavelet transform (WT), convolutional neural network (CNN), deep neural network (DNN), long short-term memory (LSTM)

## Abstract

Vehicle fault detection and diagnosis (VFDD) along with predictive maintenance (PdM) are indispensable for early diagnosis in order to prevent severe accidents due to mechanical malfunction in urban environments. This paper proposes an early voiceprint driving fault identification system using machine learning algorithms for classification. Previous studies have examined driving fault identification, but less attention has focused on using voiceprint features to locate corresponding faults. This research uses 43 different common vehicle mechanical malfunction condition voiceprint signals to construct the dataset. These datasets were filtered by linear predictive coefficient (LPC) and wavelet transform(WT). After the original voiceprint fault sounds were filtered and obtained the main fault characteristics, the deep neural network (DNN), convolutional neural network (CNN), and long short-term memory (LSTM) architectures are used for identification. The experimental results show that the accuracy of the CNN algorithm is the best for the LPC dataset. In addition, for the wavelet dataset, DNN has the best performance in terms of identification performance and training time. After cross-comparison of experimental results, the wavelet algorithm combined with DNN can improve the identification accuracy by up to 16.57% compared with other deep learning algorithms and reduce the model training time by up to 21.5% compared with other algorithms. Realizing the cross-comparison of recognition results through various machine learning methods, it is possible for the vehicle to proactively remind the driver of the real-time potential hazard of vehicle machinery failure.

## 1. Introduction

Issues such as “Metaverse”, “Big Data”, “Artificial Intelligence (AI)”, and “Digital Transformation” are in full swing [1,2], and the most critical point is the use of data acquisition (DAQ), data analysis, and machine learning, etc., to realize the integration of digitalization and smart manufacture of the system. With the popularization of 5G communication and the rapid development of Industry 4.0, the integration of technologies such as AI, Internet of Things (IoT), and cloud computing has become a very important development key in the field [3,4,5]. Recently, the scientific and technological circles are quite looking forward to realizing the integration of virtual and real (Digital Twin), thereby leading technology to another metaverse of a new digital world. These ever-changing cross-domain integrations show that the application of AI is leading the development of future technology and has penetrated into every corner.

The development of Industry 4.0 has attracted increased attention to fault diagnosis in recent years. For equipment automation, effective fault diagnosis can save time by allowing for timely remedial intervention, thus preventing potentially dangerous malfunctions [6]. Traditional fault diagnosis requires considerable personal expertise and experience, making it inefficient and costly. Expert systems present a potential solution [7]. Recently, the popularization of Internet of Things technologies has driven interest in the “Internet of Vehicles (IoV)”, in which multiple sensors are deployed on cars or other vehicles to collect timely status data [8]. Driverless vehicle applications simultaneously show to be increasingly reliable, with Tesla’s assisted driving system allowing for automatic lane changing based on real-time road conditions [9].

However, these developments are still subject to non-human-caused hazards, such as transmission failures and faulty tire conditions. In the past, such problems required passive prevention. Vehicles can fail without warning in any driving situation. Today’s vehicle diagnosis artificial intelligence technology adopts integrated signal feature extraction with various algorithm prediction models such as machine or deep learning and then uses a computer to perform rapid big data calculation and automatically classify its characteristic attributes to achieve fault prediction, identification, and diagnosis. The use of a variety of sensors and integration with the 5G communication V2X specification greatly improves the predictive ability of auto self-diagnosis. For example, in regular vehicle maintenance, the technician visually inspects the condition of the tires and transmission system using measurement devices. If the car self-diagnosis system is introduced, when various sensors of the vehicle collect the danger signal of failure or impending failure, it can immediately send a warning to the driver and the surrounding vehicles. In this way, the human resources required for car maintenance and repair will be greatly reduced, and road traffic safety will also be greatly improved.

**Figure 1:**
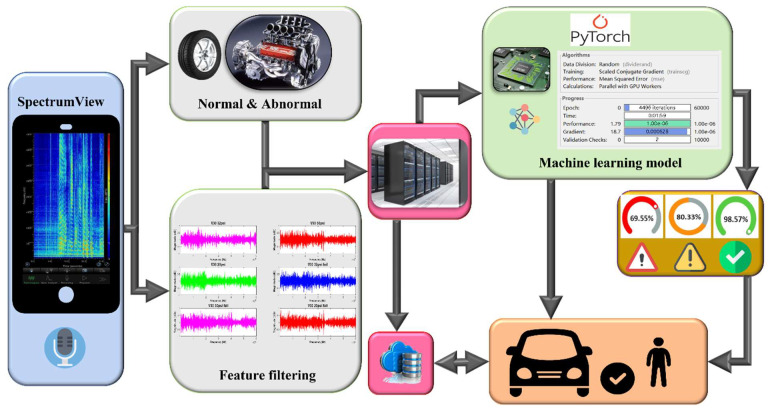
The basic procedure of an early driving fault diagnosis system and machine learning training process.

However, Zhang’s proposed car networking structure emphasizes fault prediction and maintenance [10]. Such vehicle systems rely heavily on the use of sensors to collect current fault status data and provide a timely determination of the fault location. Such data can be transmitted to a nearby service station through the cloud for initial diagnosis, thus greatly reducing repair times, as shown in Figure 1. Moreover, advances in car sharing technology [11] now allow for warnings to be transmitted to neighboring vehicles. To date, many fault identification solutions have been proposed, such as the Distributed Fiber-Optic Acoustic Sensing (DAS) system proposed by Li et al. to detect vehicle vibrations on the road in order to identify vehicle model and know its amount [12], or to identify specific distortions and locate faulty turn phases [13]. The performance of AI approaches for diagnostics [4] relies on data quality and quantity. Currently, the two main directions for data sources are audio and video. In 2018, the BMW began to introduce AI-based image recognition techniques to inspect automotive sheet metal quality. Meanwhile, voice recognition technology has been widely deployed, with applications such as Siri and Google voice assistants continuously collecting longitudinal data to achieve high degrees of recognition accuracy.

Currently, no mature technology exists for vehicle voiceprint recognition, and the present research focuses on the development of voice recognition applications for use in vehicles. Feature extraction is the key aspect in machine learning, and good features selection can significantly improve model training accuracy. When an object vibrates freely, it has a fixed specific frequency and mode. As long as the rigidity, structure, and shape of these objects are fixed, the natural frequency of the vibration is fixed. As a result, it will not change with time and the external force environment. In this study, sensors are used to collect signals and analyze the early fault characteristics of rotating machinery to identify the signals. Typically, signals are obtained from non-stationary and non-linear machines with a certain degree of noise [14], which may obscure important fault features. Such signals, such as cancer, cannot be clearly identified initially and are often obscured by complex background environmental noise. If the weak abnormal signal in the early stage of the fault cannot be captured, the opportunity for early maintenance will be missed, resulting in vehicle accidents. In this study, a more accurate sensor is used to obtain the abnormal signal generated by the initial failure of the vehicle. The non-contact measurement method of a MEMS microphone array is adopted, because the acoustic microphone has high resolution in the middle and high frequency bands. The fault characteristics can be presented by the mid-to-high frequency signal in the early stage of the fault [15,16], and consequently, the acoustic sensing technology is very suitable for the early abnormal detection of the rotating mechanical system.

Instead of using the MEMS microphone array, addressing these problems requires the use of appropriate filtering methods, and methods such as the Mel-scale frequency cepstral coefficient (MFCC) [17,18], Fast Fourier transform (FFT) [19,20], order-tracking technology [16] and wavelet transform [21] have been applied to machinery fault diagnosis. Linear prediction coefficients (LPC) have been applied to many modern speech processing systems for applications including coding, synthesis, analysis and recognition [22]; the initial model is constructed using historical data, and new data testing and verification can be used to predict the associated outcomes of audio signal data. Previous studies have used LPC to achieve perfect fault diagnosis performance [23], and compared with the above-mentioned filtering algorithm, LPC uses less resources to achieve high-resolution spectra. The development of artificial intelligence (AI) and the Internet of things (IoT) [4,24] has raised new possibilities. For example, failure detection of vehicle suspension systems [25] uses AI to achieve early prediction, thus reducing the occurrence of vehicle accidents.

Machine learning algorithms can be used to perform large-scale data analysis. Support vector machine (SVM) was first used for fault diagnosis in the late 1990s [26]. Artificial neural networks (ANN) are among the most widely used methods for fault diagnosis and have been used for mechanical fault prediction [27] and the later development of multilayer neural networks [28]. The improvement of hardware capabilities further drove the application of neural networks. Increasing the number of neurons deepens the hidden layer, thus improving the recognition rate. Deep neural networks (DNN) are still in the development stage for fault identification applications, and many challenges still need to be addressed. For example, very large amounts of data can significantly impair processing efficiency [29]. Even with strong hardware support, data processing presents a major challenge and is difficult to apply in practice. This article also discusses the challenges of finding a suitable activation function to accelerate neuron convergence. Previously, CNNs were mainly used for image and facial recognition [30,31] and have rarely been used for classification in speech processing. In this experiment, the spectrum dataset is classified using a CNN model for comparison with different types of classifiers, such as DNNs. The remainder of this article is as follows. Section 2 discusses the theoretical background, including LPC, Wavelet, DNN, CNN, and LSTM algorithms. Section 3 introduces the experimental framework, collects sound signals, and converts them into voiceprint characteristic spectra to build a dataset. Section 4 introduces the test results of ML methods on the dataset. Section 5 draws conclusions and presents directions for future work.

## 2. Method Theory

### 2.1. Linear Predictive Coding Method

**Figure 2:**
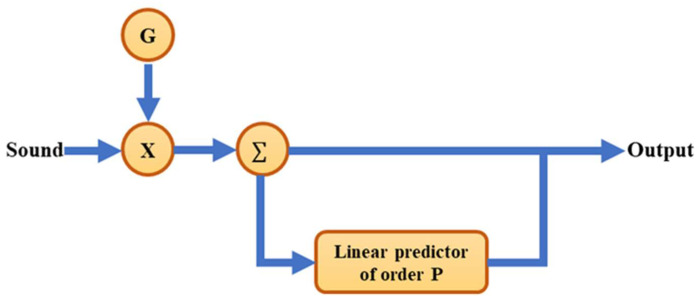
The spectral features filtered by LPC method.

Linear prediction coefficient (LPC) is one of the most effective speech analysis techniques and is widely used in speech recognition and audio compression [32], as illustrated in Figure 2, and G is gain value. LPC can provide accurate speech parameter prediction, making it well suited for modeling the transfer characteristics of sound sources. Good analytical performance is also observed in the extraction of noise characteristics of the mechanical transmission system when using LPC. The main theory is that the input *x*(*k*) of a linear discrete-time system is a linear weighted combination of the input samples and the output of previous samples. The following function can be written:

(1) $$x\left(k\right)=\overset{p}{\underset{i=1}{\sum }}a_{i}\cdot x\left(k-i\right)+e\left(k\right)$$

where integer k is the time index, $\alpha_{i}$ is defined as the linear prediction coefficient, and *p* represents the past coefficient. Given a prediction signal x(k), the number of prediction errors e(k) is given by:

(2) $$e\left(k\right)=x\left(k\right)-\hat{x}\left(k\right)=x\left(k\right)-\overset{p}{\underset{i=1}{\sum }}a_{i}\cdot x\left(k-i\right)$$

$\hat{x}\left(k\right)$ is the prediction sample, and $\alpha_{i}$ is determined by e(k) to minimize the mean square error (MSE). The equation is:

(3) $$a_{i}=E\left[e^{2}\left(k\right)\right]$$

### 2.2. Wavelet Transform (WT)

The Shannon recovery formula can assist in restoring the original analog function y(t), where the relationship can be as follows

(4) $$y\left(t\right)=\underset{n\epsilon Z}{\sum }x\left(nh\right)\frac{\sin\pi \left(t-nh\right)}{\pi \left(t-nh\right)}$$

The continuous wavelet transform (CWT) of the time-domain signal y(t) can be expressed by the following transformation formula:

(5) $$W_{\emptyset }x\left(b,a\right)=\frac{1}{\sqrt{a}}\int_{-\infty }^{\infty }y\left(t\right)\bar{\emptyset \left(\frac{t-b}{a}\right)}dt\;$$

If $a=\frac{1}{2^{\sigma }}$ is used as the scale, $b=\frac{k}{2^{\sigma }}$ is used as the translation, and both s and k are integers, in the time-scale plane, the CWT of *y*(*t*) is a value in ($\frac{k}{2^{\sigma }}$, $\frac{1}{2^{\sigma }}$), which represents the relationship between x(t) and $\bar{\emptyset }\left(t\right)$ at that time-scale point, and is called discrete wavelet transform (DTW). This method generates a set of sparse values on the time-scale plane. The expression is as follows:

(6) $$w_{k,s}=W_{\emptyset }x\left(\frac{k}{2^{\sigma }},\frac{1}{2^{\sigma }}\right)=\int_{-\infty }^{\infty }y\left(t\right)\bar{\emptyset \left(\frac{t-\frac{k}{2^{\sigma }}}{\frac{1}{2^{\sigma }}}\right)}dt$$

With this expression, the wavelet coefficient can be represented in $\left(b=\frac{k}{2^{\sigma }},\;a=\frac{1}{2^{\sigma }}\right)$. That is the mapping to the time-domain signal y(t) under the discrete-time scale [33].

### 2.3. Deep Neural Network (DNN)

**Figure 3:**
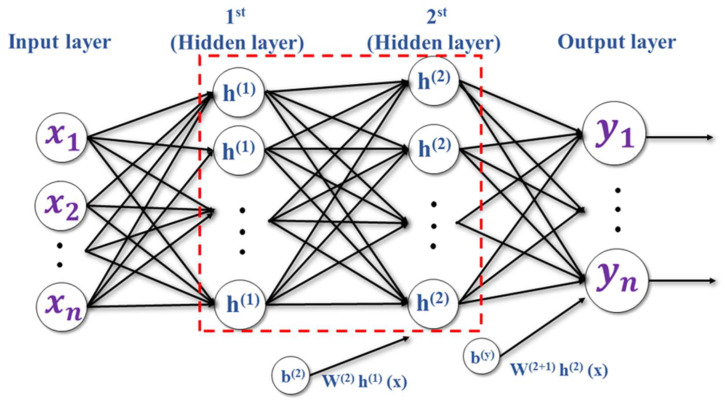
General MLP neural network architecture for the automotive.

DNN is an artificial neural network used for supervised learning. Neural networks make “judgments” by simulating the operation of neurons in human brain cells. Such networks contain many computational layers, using the input layer and output layer as perceptrons, and with one or more hidden layers between them. Networks with multiple hidden layers are called deep neural networks (DNNs). Using a large number of hidden layer training data can help improve the accuracy of the weight value classification. The activation function plays an important role in neural networks. It can make neurons improve gradient descent performance through nonlinear conversion. Different activation functions can be used to improve the MLP performance [34]. Figure 3 shows MLP structure with two hidden layers. $W^{\left(m\right)}$ is defined as weighted, which connects between the hidden layers. $b^{\left(m\right)}$ is basis of the *m*th layers (m>0). $a^{\left(m\right)}\left(x\right)$ indicates the previous level $h^{\left(m-1\right)}$ and $W^{\left(m\right)}$ are multiplied and added to b(m). The value of $a^{\left(k\right)}\left(x\right)$ is inserted into the activation function g(x), and the result is the output $y_{n}$ [17].

(7) $$a^{\left(m\right)}\left(x\right)=b^{\left(m\right)}+W^{\left(m\right)}h^{\left(m-1\right)}\left(x\right)$$

For the *i*th neuron in the *m*th hidden layer, the concept is equated as below:

(8) $$h^{\left(m\right)}{\left(x\right)}_{i}=g\left(a^{\left(m\right)}{\left(x\right)}_{i}\right)$$

The equation of the desired output layer is formulated as:

(9) $$y_{n}=g\left(a^{\left(m+1\right)}\left(x\right)\right)=f\left(x\right)$$

### 2.4. Convolutional Neural Network (CNN)

**Figure 4:**
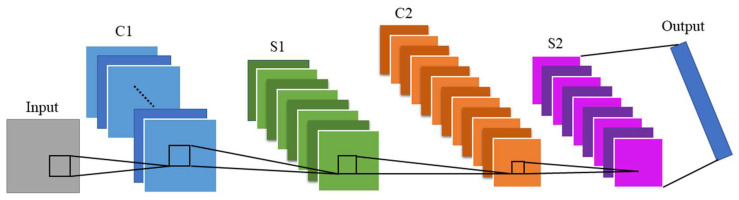
Architecture of the CNN.

The basic CNN architecture was first proposed in 1980 by Kunihiko Fukushima [35]. Its structure was inspired by the concept of simple and complex cells in the brain’s visual cortex [36], as an extension of the ANN architecture. A CNN is composed of a convolution layer, a pooling layer, and a fully connected layer. The convolutional layer is a feature map obtained by applying the summing of the product of the input pixels. The pooling layer is used to reduce the feature dimensionality of the input, thus preventing overfitting. Finally, the fully connected layer flattens the features into a one-dimensional vector for classification. Some well-known CNN models, such as AlexNet [30], GoogLeNet [37], VGGNet [38], LeNet-5 [39], etc., have been widely used in image recognition. Among these, a CNN block diagram has been successfully applied for image-based fault diagnosis [40]. Figure 4 shows the CNN architecture, which is applied here to identify audio signal features in vehicles.

### 2.5. Long Short-Term Memory (LSTM)

**Figure 5:**
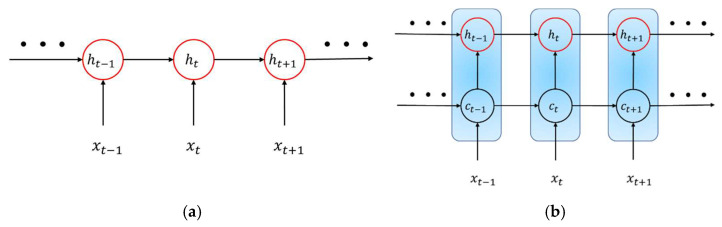
Architecture of RNN. (**a**) Normal architecture of RNN. (**b**) LSTM network.

In a general recurrent neural network, there is only one hidden state unit $h_{t}$, and the parameters of the hidden state unit at different times are the same, as shown in Figure 5a. This makes the recurrent neural network a long-term dependence problem that can only be sensitive to short-term input. LSTM adds a cell state unit $c_{t}$ on the basis of an ordinary recurrent neural network, which has variable connection weights at different times to solve the problem of gradient disappearance or gradient explosion in an ordinary recurrent neural network, as shown in Figure 5b. In Figure 5, $h_{t}$ is the hidden state unit (short-term state unit), and $c_{t}$ is the unit state unit (long-term state unit), which together constitute the LSTM architecture [41].

Unlike general recurrent neural networks, LSTMs reference gating units. Gating is the unit learned by the neural network to control the storage, utilization, and discarding of signals. For each time t, LSTM has three gating units: input gate $i_{t}$, forget gate $f_{t}$ and output gate $o_{t}$. The input of each gating unit contains the sequence information $x_{t}$ at the current moment and the hidden state unit $h_{t-1}$ at the previous moment. The actual calculation formula is:

(10) $$i_{t}=\sigma \left(W_{i}x_{t}+U_{i}h_{t-1}+b_{i}\right)$$

(11) $$f_{t}=\sigma \left(W_{f}x_{t}+U_{f}h_{t-1}+b_{f}\right)$$

(12) $$o_{t}=\sigma \left(W_{o}x_{t}+U_{o}h_{t-1}+b_{o}\right)$$

Among them, W and U are the weight matrices, b is the bias vector, and σ(·) is the startup function. It can be found that the calculation methods of the above three gating units are the same (all equivalent to a fully connected hierarchy), and only the weight matrix and the bias vector are different. The setting value range of the startup function σ(·) is generally [0, 1], and the commonly used startup function is the sigmoid function.

By multiplying the gating unit and the signal data element by element, the amount of information to be retained after the signal passes through the gating can be controlled. For example, when the state of the gate unit is 0, the signal will be completely discarded; when the state is 1, the signal will be fully retained; and when the state is between 0 and 1, the signal will be partially reserved.

**Figure 6:**
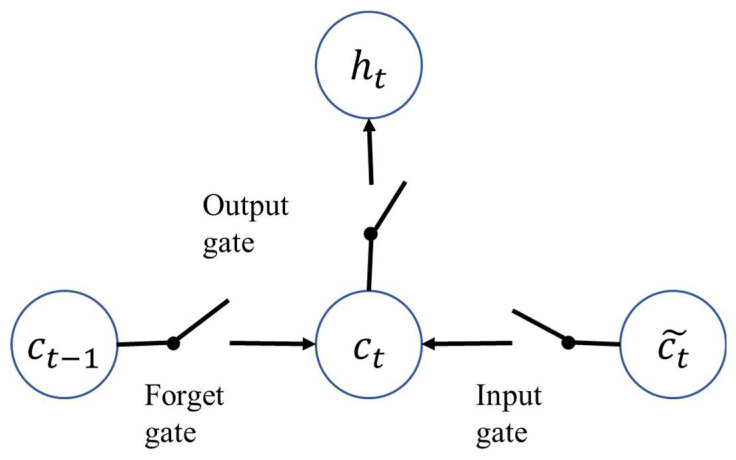
Schematic of LSTM cell.

LSTM operates by using three gating units and cell state units. Figure 6 is a schematic diagram of the gating unit and state unit in LSTM. It can be seen that the transmission of the cell state unit from $c_{t-1}$ at the previous moment to $c_{t}$ at the current moment is jointly controlled by the input gate and the forgetting gate. The input gate determines how much of the input information $\tilde{c_{t}}$ is absorbed at the current moment. The forget gate determines how much of the cell state unit $c_{t-1}$ is not forgotten at the previous moment, and the final cell state unit $c_{t}$ is generated by the sum of the two gated signals. The actual formula is:

(13) $$\tilde{c_{t}}=\tanh\left(W_{c}x_{t}+U_{c}h_{t-1}+b_{c}\right)\;$$

(14) $$c_{t}=\left(f_{t}⊙c_{t-1}\right)⊕\left(i_{t}⊙\tilde{c_{t}}\right)\;$$

Among them, ⊙ is the element-wise dot product operation. The hidden state unit $h_{t}$ of LSTM is determined by the output gate and $c_{t}$:

(15) $$h_{t}=o_{t}⊙\tanh\left(c_{t}\right)$$

**Figure 7:**
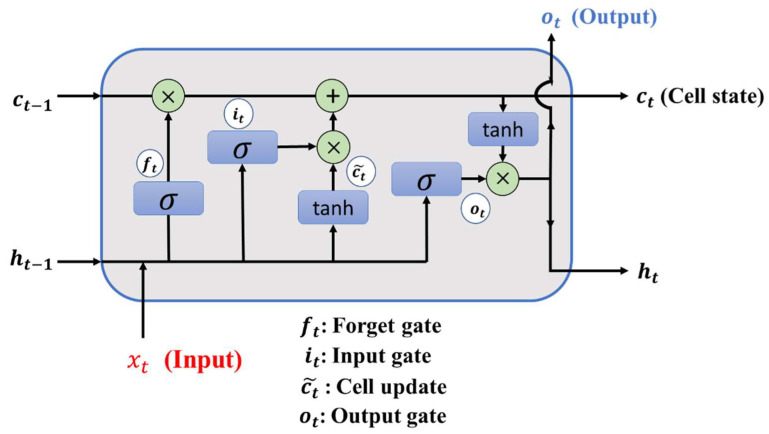
Complete architecture of LSTM.

It can be seen that in LSTM, not only the hidden state unit $h_{t-1}$ and $h_{t}$ have a relatively complex cyclic connection, but also the internal unit state unit $c_{t-1}$ and $c_{t}$. There is also a linear self-circulating relationship between them. The linear self-loop between cell state units can be seen as sliding to process information at different times. When the gated unit is on, the past information is remembered; when the gated unit is off, the past information is discarded. On the whole, LSTM provides a path for the long-distance continuous circulation of gradients through the linear self-circulation of the gating unit and the cell state unit, which changes the propagation mode of information and gradients in the previous recurrent neural network and solves the long-term dependency problem. The complete LSTM architecture is shown in Figure 7.

## 3. Experimental Structure

**Figure 8:**
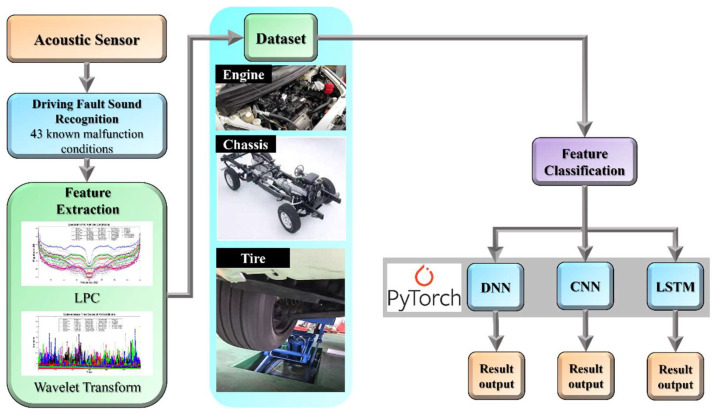
Systematically experimental structure with the use of classifying methodology for a driving fault acoustic signal system.

This research is divided into three parts. Figure 8 shows the structure of the vehicular audio signal diagnosis experiment. The first part focuses on signal characteristic filtering. We use acoustic sensors to collect 43 vehicle fault signals. Table 1 shows the 43 fault conditions, including 18 different types for the tires, 6 types for the belt, 16 types for the chassis, and 3 types for the engine.

**Figure 9:**
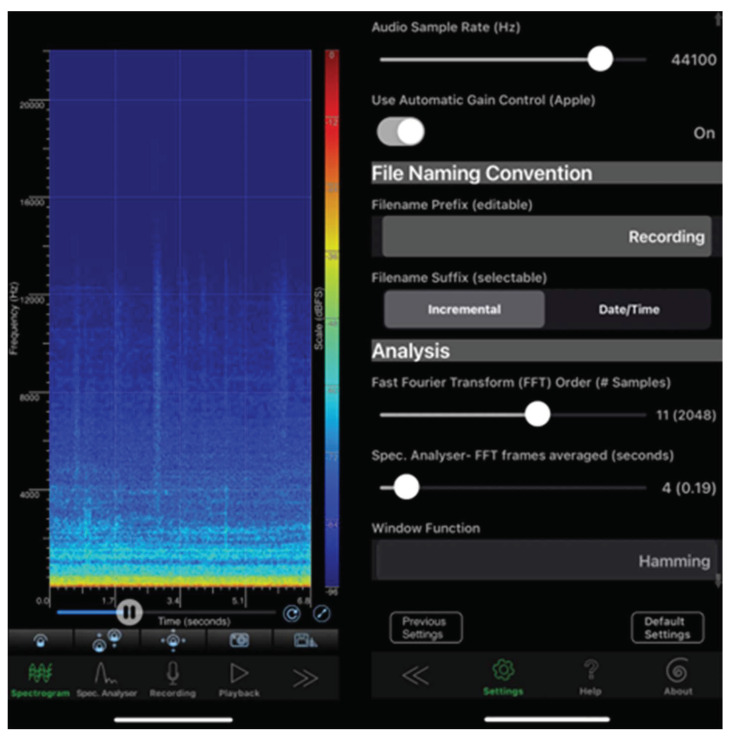
Hamming window function by the measurement industrial fault signals.

To obtain a considerable amount of the fault signals, the IoT device architecture plays an important role in ensuring that a large number of faulty signal samples are obtained. The combined equipment and network characteristics enable us to obtain a dynamic time signal and convert the energy spectrum on the experimental equipment. The Hamming window function is used to obtain the spectrum signal. Figure 9 shows the settings of the spectrum signal.

**Figure 10:**
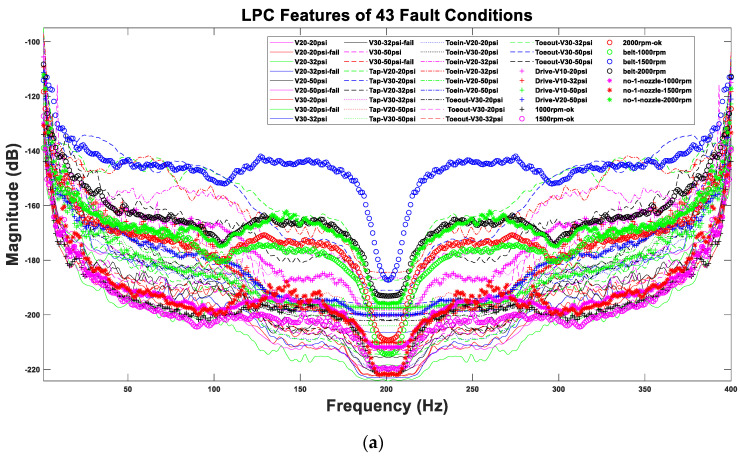

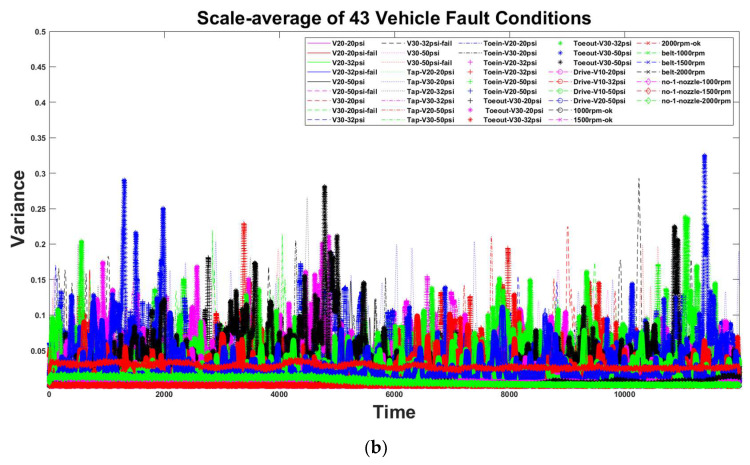
Features of the 43 conditions of the acoustic signals after (**a**) LPC and (**b**) wavelet extractions.

The sampling frequency is 44,100 Hz, and the acquisition time of each data sample is 40 s. In converting the frequency spectrum, signal preprocessing is first performed to eliminate background noise. According to the “Nyquist–Shannon” sampling theorem, the sampling frequency must be greater than twice the maximum frequency required for reproduction [42]. Since the human hearing range is approximately 20–20,000 Hz, the sampling frequency must be greater than 40 kHz. The audio codec of our smartphone has a standard sampling frequency of 44.1 kHz, which corresponds to a sampling rate of 20–20 kHz in the audible range of the human ear [43]. This analysis uses the LPC and wavelet algorithms for filtering, and the sound signal is converted from a continuous time domain signal to a sound spectrum frequency domain. Sound features are filtered through MATLAB to create a training dataset. Figure 10 shows the 43 normal and fault conditions of LPC and wavelet on the MATLAB platform with audio signal spectrum characteristics.

**Figure 11:**
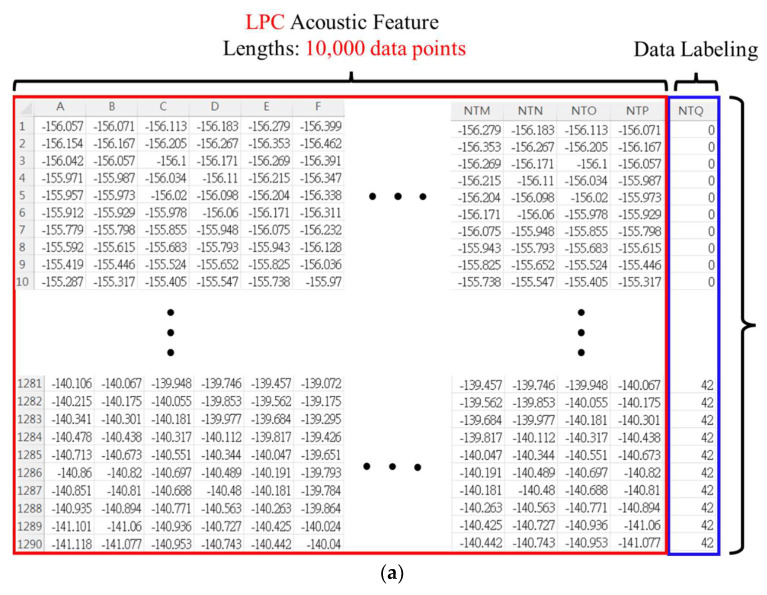

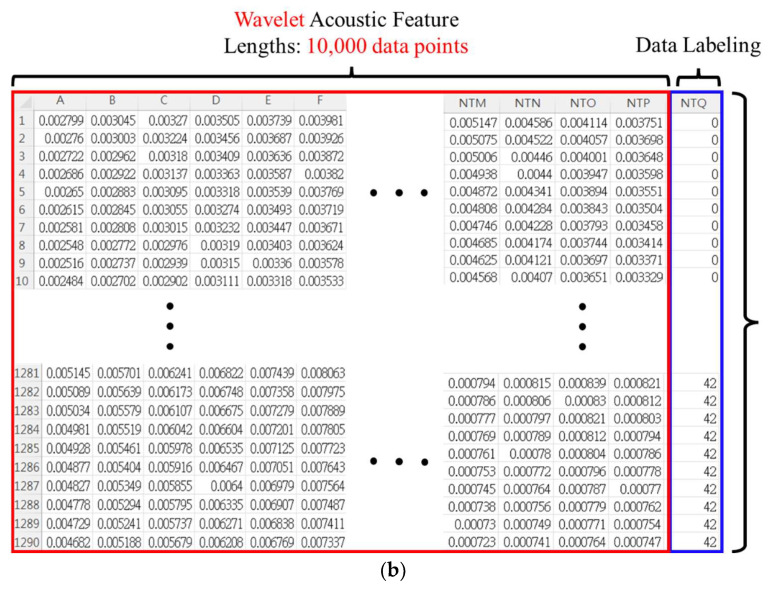
Dataset map. The horizontal axis is the data labeling of the audio signal spectrum, and the vertical axis is the predictive coefficient number sequence. (**a**) LPC and (**b**) wavelet.

The second part establishes the spectral characteristic signal dataset. To effectively identify the characteristics of various types of faults, the original spectral characteristics are reduced to 10,000 characteristic lengths as filtered spectral characteristics. Then, 30 and 40 sets of characteristic coefficients were set for 43 fault conditions. A total of 1290 and 1720 voiceprint characteristic data were constructed, respectively. The voiceprint feature on the horizontal axis is expected to be set to 10000 points, that is, 10000 pieces of feature data to train the model. The dimensions of the total dataset are 1290*10000 and 1720*10000, respectively. Figure 11 shows the settings diagram of the dataset.

In the third part, after completing the construction and labeling of the dataset, the Pytorch architecture in Python is used to import the dataset into the three algorithms of DNN, CNN and LSTM for classification. The Pytorch architecture used in this study uses adaptive moment estimation (Adam) as the optimizer function. The Adam is an adaptive learning rate algorithm whose essence is an RMSprop optimization method with a momentum term and is currently the most widely used model training optimizer [44]. The experimental process is mainly used to cross-compare the datasets constructed by two different filtering algorithms LPC and wavelet and the differences between the recognition results and learning speeds caused by the three machine learning algorithms. The learning rate and batch size are based on the premise of using the least Epoch (the number of iterations) to adjust the parameters to achieve the best recognition rate.

The learning rate controls the learning rate of the model. The larger the learning rate, the faster the convergence rate and the less training time. After the extreme value is exceeded, the loss function stops decreasing and oscillates at a certain position. The smaller the learning rate, the slower the convergence speed, the more time it takes to train the model, and the easier the network to enter the local minimum, which makes the loss function converge poorly. Therefore, the appropriate learning rate can be adjusted by observing the change of the model loss parameters. In this study, we set the learning rate of the three algorithms to 0.00001 after many experiments, which not only achieves the best model convergence overall, but also facilitates cross-validation of different deep learning algorithms.

**Figure 12:**
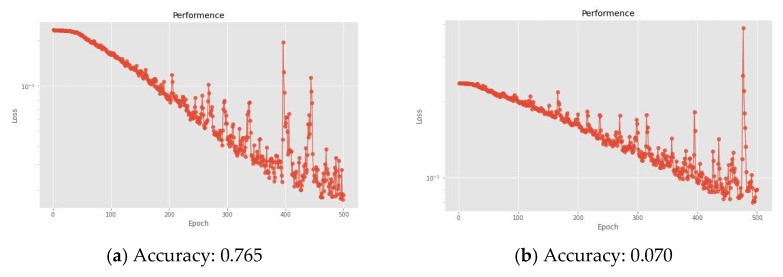
Loss function and accuracy of various hidden layer settings of DNN: (**a**) 12 layers. (**b**) 20 layers.

In terms of the hidden layer setting of the DNN algorithm in this study, at first, we tried to use a DNN architecture with 12 hidden layers, but it was found that the training results of the model with too few hidden layers were not ideal, as shown in Figure 12a. After that, we used a DNN architecture with 20 hidden layers and found that there was an overfitting problem. In addition, the model training and identification results were also extremely poor, as shown in Figure 12b. Therefore, it was finally decided to adopt a DNN architecture with 15 hidden layers as the hidden layer parameter setting in this study.

**Figure 13:**
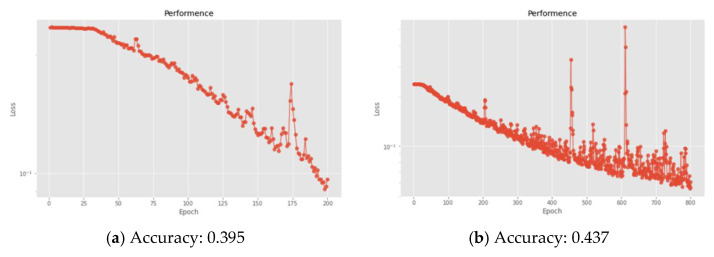
Loss function and accuracy of various epoch settings of DNN. (**a**) Epoch = 200, underfitting. (**b**) Epoch = 800, overfitting.

Taking the LPC 30 feature as an example, if epoch = 200, the number of iterations of the model training is insufficient, and underfitting occurs. Although the model training time can be the shortest, the recognition accuracy is low (as shown in Figure 13a). If we lengthen the epoch to 800, the model training stability is extremely low, and overfitting occurs (as shown in Figure 13b). Therefore, it is more appropriate to use epoch = 500, thereby avoiding overfitting and underfitting. In addition, in order to facilitate the comparison of the learning effects of the three deep learning algorithms DNN, CNN and LSTM on the same dataset, we use the same epoch to facilitate cross-comparison of the time consumed by the training model.

**Figure 14:**
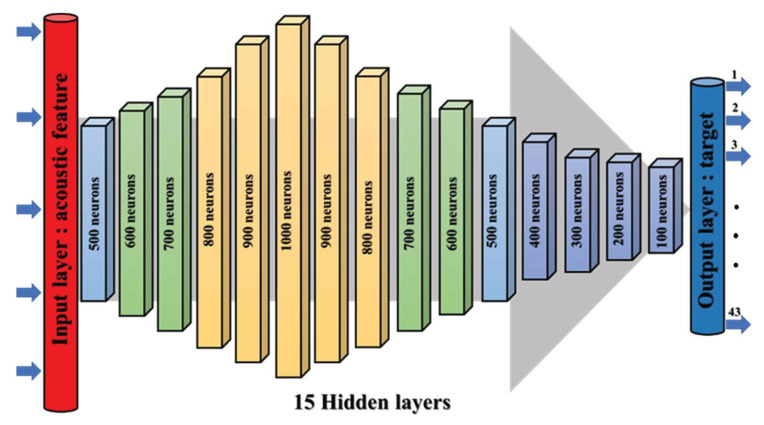
DNN architecture.

**Figure 15:**
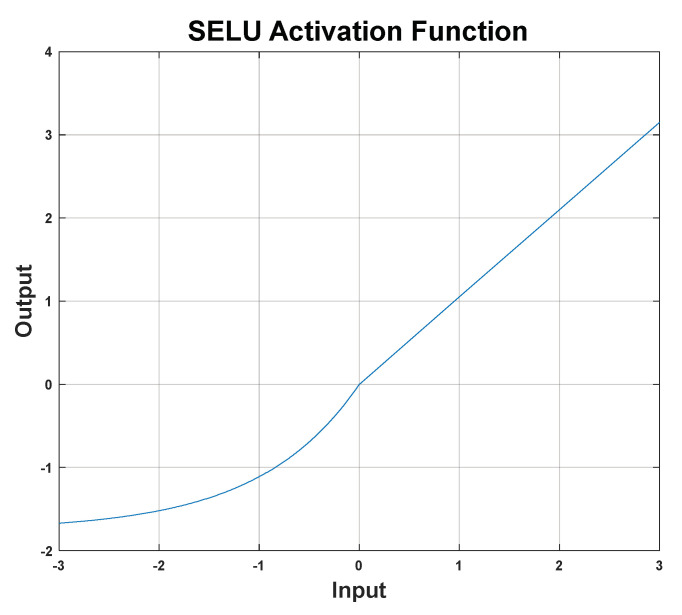
SELU activation function.

The DNN parameters of this experiment are set as: the learning rate = 0.00001, the iteration time (Epoch time) = 500, the batch size (Batch) = 128, the test size = 0.3. We adopted a 15-layer deep neural network architecture, and the number of neurons in each hidden layer is shown in Figure 14. The connection between the hidden layers is fully connected. In order to solve the problem of gradient disappearance at saturation, Xavier Glorot et al. proposed a linear rectified function (Rectified Linear Unit, Relu) [45]. The disadvantage is that when the variable is updated too fast and when the function has not found the optimal value, the neuron will become less than 0 and the neuron will die. Therefore, the activation function we selected in the experiment is Selu (Scaled Exponential Linear Unit), and Selu is a variant of Relu [46], as shown in Figure 15, where its function is:

(16) $$Selu\left(x\right)=\lambda \{\begin{array}{ll} x, & x>0\\ \alpha \left(e^{x}-1\right), & x<0 \end{array},$$

where λ is a fixed value of 1.05070098736, and α is 1.67326324235.

**Figure 16:**
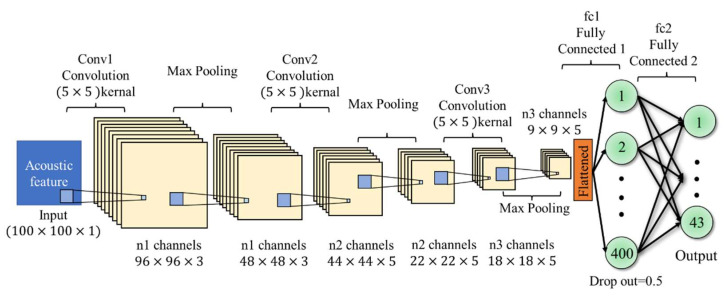
CNN architecture.

Furthermore, in this experimental CNN model, three convolutional layers and three pooling layers are used to reduce the size of audio signal features. The sizes of the three convolutional layers are (5×5×3), (5×5×5), (5×5×5) in sequence, and the pooling layers are all 2×2. That is, the filter measures is 2×2, as shown in Figure 16. The network architecture parameter settings include a learning rate of 0.00001, a batch size of 128, and a test set scale of 0.3.

**Figure 17:**
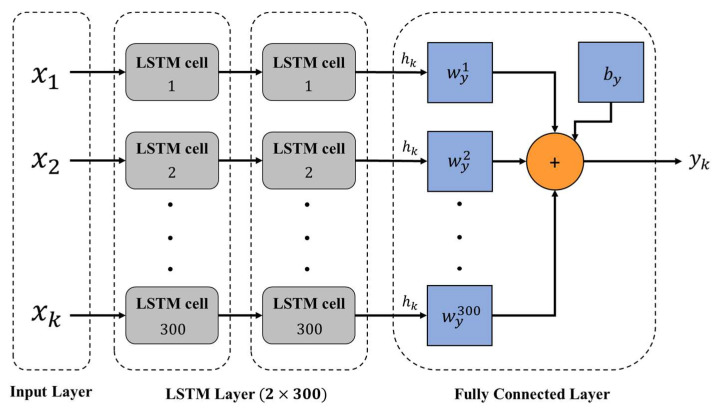
LSTM architecture.

The third neural network approach used in this experiment is the Long Short-Term Memory (LSTM) algorithm. The LSTM architecture we used in the experimental process is based on two hidden layers of the network, and 300 hidden neurons are used in each fault condition with 30 and 40 features, where the LSTM training model network label layer is 2×300, indicating that there are two hidden layers containing 300 hidden neurons, as shown in Figure 17. Here, we compare the training performance of the LPC dataset and the wavelet dataset. Two different spectral datasets use a batch size of 128, the learning rate is set to 0.00001, and the number of iterations is set to 500.

## 4. Results and Discussion

This part uses the DNN algorithm to model the training dataset in the Python Pytorch framework. We compare the results for DNNs with more than 10 hidden layers. Generally, in deep learning, deeper hidden layers are more accurate than shallow hidden layers.

As showed in Table 2, the LPC dataset uses the CNN algorithm to achieve better identification results, and the LSTM algorithm is extremely poor, followed by DNN. The wavelet dataset using LSTM and DNN has a good identification effect, while the effect of the CNN algorithm is slightly worse. In terms of wavelet dataset, the identification accuracy of the DNN algorithm is as high as 1.00, which is 16.57% higher than 0.86 of the CNN algorithm and 13.82% higher than 0.88 of the LSTM algorithm. As far as the LPC dataset is concerned, the accuracy rate of the CNN algorithm reaches 1.00, which is 72.77% higher than that of the DNN algorithm. In terms of model training time, the training time of the two datasets imported into the CNN algorithm is the longest, followed by DNN, and the shortest by LSTM. Compared with the LPC dataset, the wavelet dataset takes a longer time to import the three machine learning algorithms for the model training, and the difference is the largest in the CNN algorithm, which is 3.13% longer than the LPC dataset training time.

**Figure 18:**
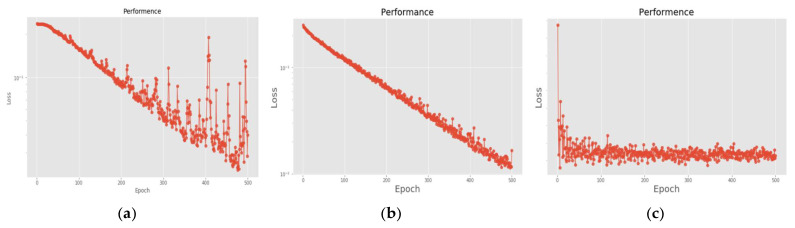
Loss function of LPC 30 feature dataset. (**a**) LPC + DNN. (**b**) LPC + CNN. (**c**) LPC + LSTM.

**Figure 19:**
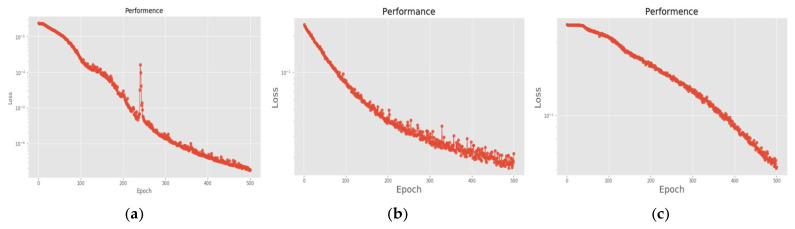
Loss function of wavelet 30 feature dataset. (**a**) Wavelet + DNN. (**b**) Wavelet + CNN. (**c**) Wavelet + LSTM.

**Figure 20:**
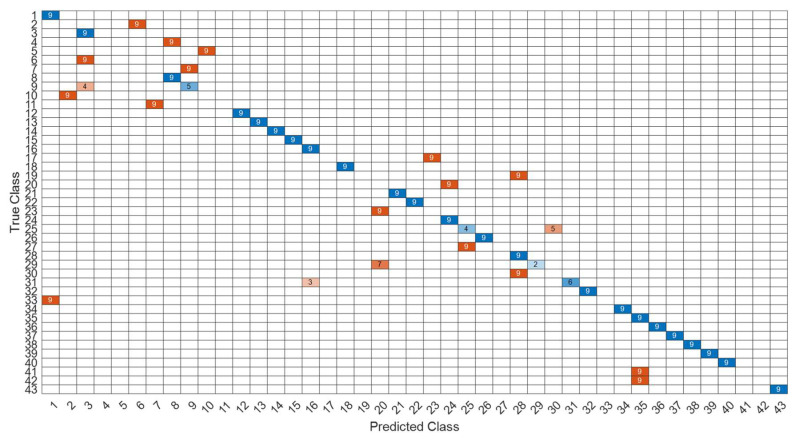
The confusion matrix of the DNN model with LPC 30-features dataset.

**Figure 21:**
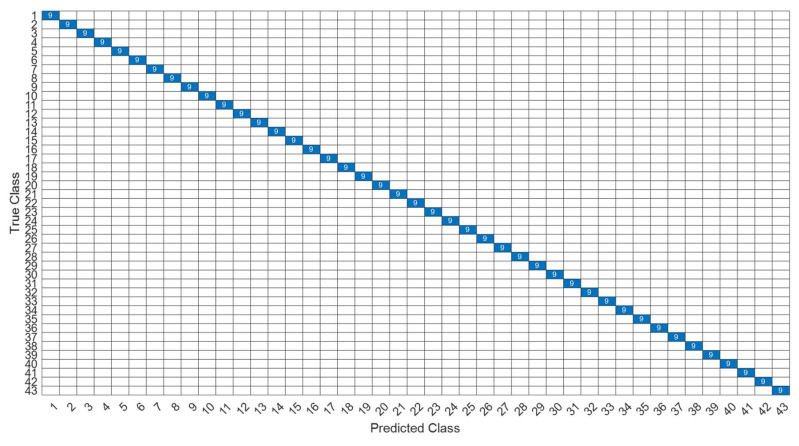
The confusion matrix of the CNN model with LPC 30-features dataset.

**Figure 22:**
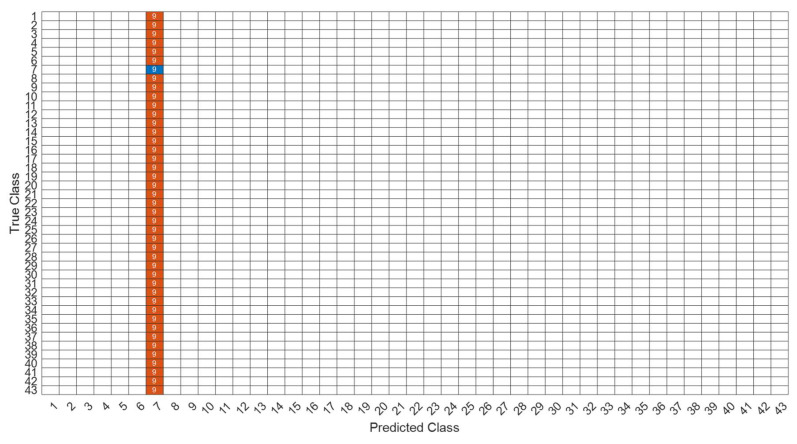
The confusion matrix of the LSTM model with LPC 30-features dataset.

**Figure 23:**
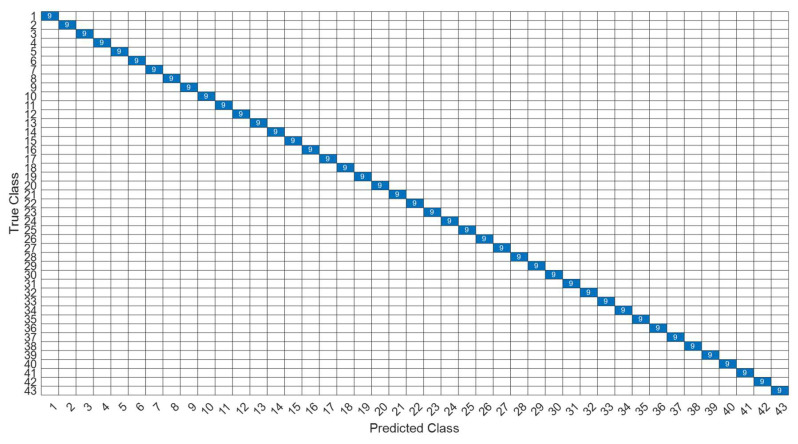
The confusion matrix of the DNN model with wavelet 30-features dataset.

**Figure 24:**
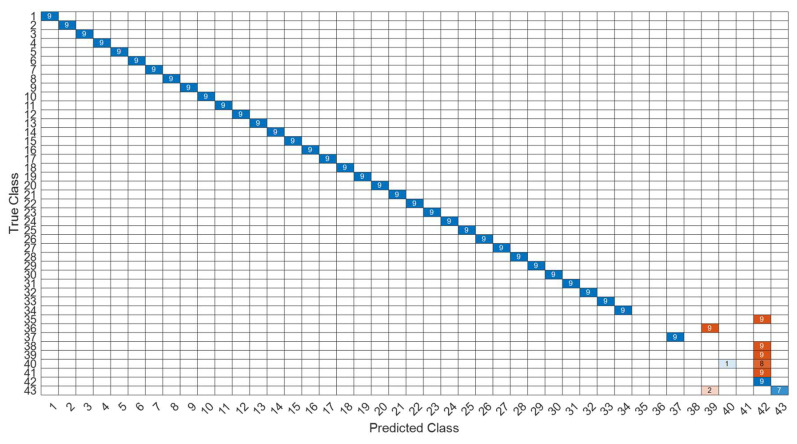
The confusion matrix of the CNN model with wavelet 30-features dataset.

**Figure 25:**
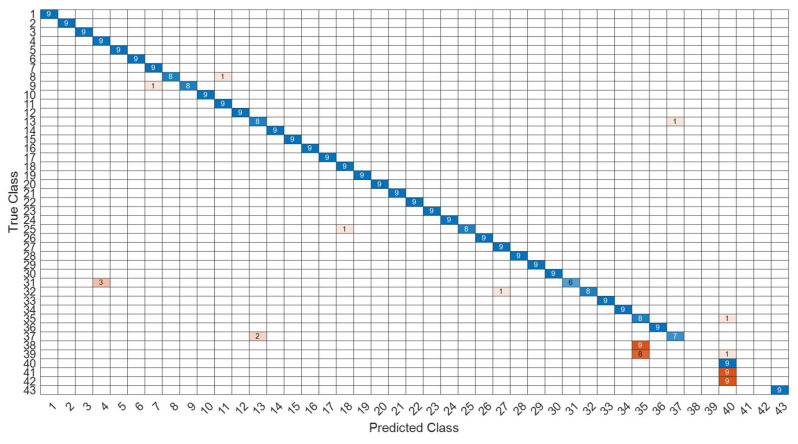
The confusion matrix of the LSTM model with wavelet 30-features dataset.

Figure 18 is a comparison of the loss functions of the LPC feature dataset (with 30 features). Figure 18a–c are the loss functions using DNN, CNN and LSTM algorithms, respectively. We found that the convergence speed of DNN and CNN is faster. Compared with the wavelet dataset, the CNN algorithm converges more stably, whereas LSTM has extremely poor convergence here. Figure 19 shows the comparison of the loss function of 30 kinds of features by wavelet. Figure 19a–c are the loss functions using DNN, CNN and LSTM algorithms, respectively. We found that DNN and CNN converge faster, but DNN is more stable overall. Furthermore, the gradient convergence of LSTM is slower, but the stability is higher than the previous two. In addition, Figure 20, Figure 21 and Figure 22 are the confusion matrices of the LPC datasets with 30 features imported into DNN, CNN and LSTM algorithms for classification and identification; Figure 23, Figure 24 and Figure 25 are the wavelet datasets of 30 features imported into DNN and CNN confusion matrix for classification and identification with LSTM algorithm. The results of our experiments can also be seen from the confusion matrix.

Table 3 presents the results with three different deep learning algorithm of 40-feature datasets. Similar to taking 40 sets of Cdelta coefficients and 30 sets of P coefficients for each fault condition, the LPC dataset uses the CNN algorithm to achieve better identification results, and the LSTM algorithm is extremely poor, followed by DNN. The wavelet dataset using LSTM and DNN has a good identification effect, while the effect of the CNN algorithm is slightly worse. For the wavelet dataset, the identification accuracy of the DNN algorithm and the LSTM algorithm is as high as 1.00, which is 9.55% higher than the 0.86 of the CNN algorithm. As far as the LPC dataset is concerned, the accuracy rate of the CNN algorithm reaches 1.00, which is 20.84% higher than that of the DNN algorithm. In terms of the model training time, the training time of the two datasets imported into the CNN algorithm is the longest, followed by LSTM. The DNN is the shortest in the training time. Unlike the 30 features, DNN is a calculus with a shorter training time between the two. Compared with the LPC dataset, the wavelet dataset has a shorter time for model training when the three machine learning algorithms are applied.

**Figure 26:**
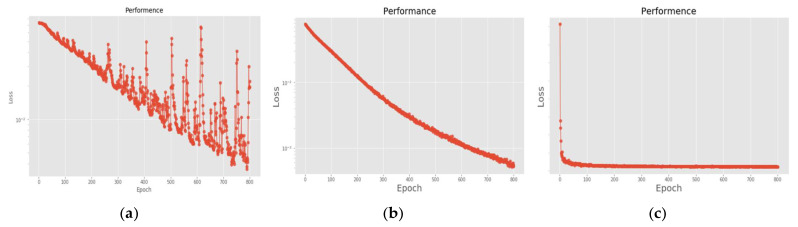
Loss function of LPC 40-features dataset. (**a**) LPC + DNN. (**b**) LPC + CNN. (**c**) LPC + LSTM.

**Figure 27:**
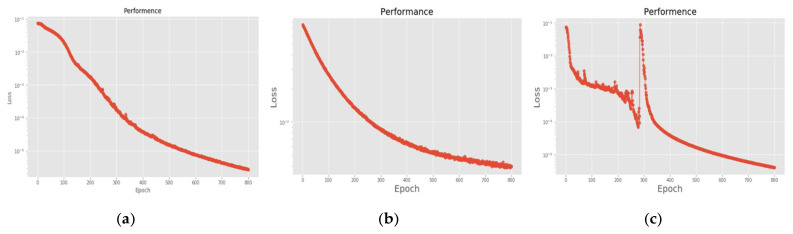
Loss function of wavelet 40-features dataset. (**a**) Wavelet + DNN. (**b**) Wavelet + CNN. (**c**) Wavelet + LSTM.

**Figure 28:**
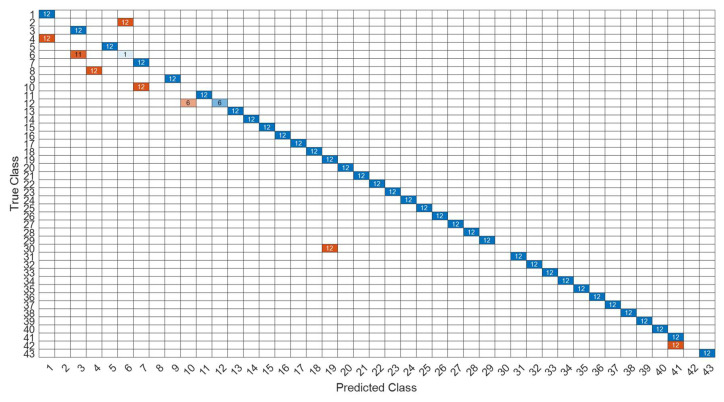
The confusion matrix of the DNN model with LPC 40-features dataset.

**Figure 29:**
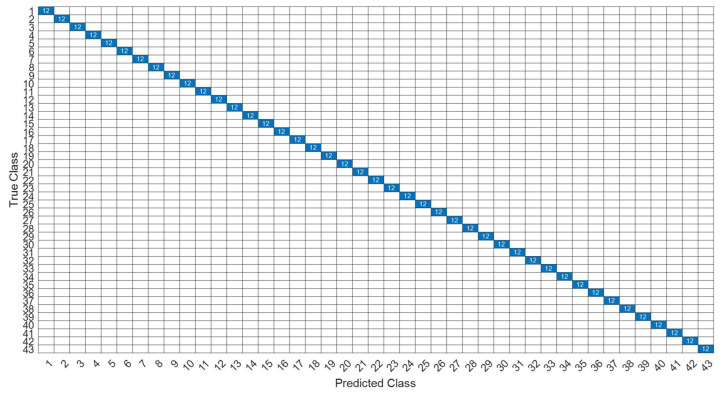
The confusion matrix of the CNN model with LPC 40-features dataset.

**Figure 30:**
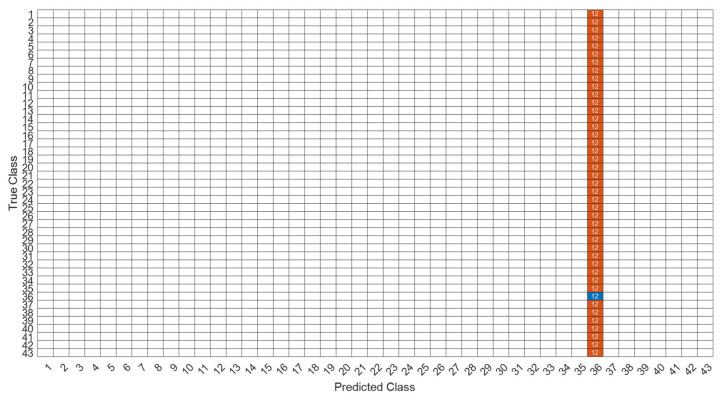
The confusion matrix of the LSTM model with LPC 40-features dataset.

**Figure 31:**
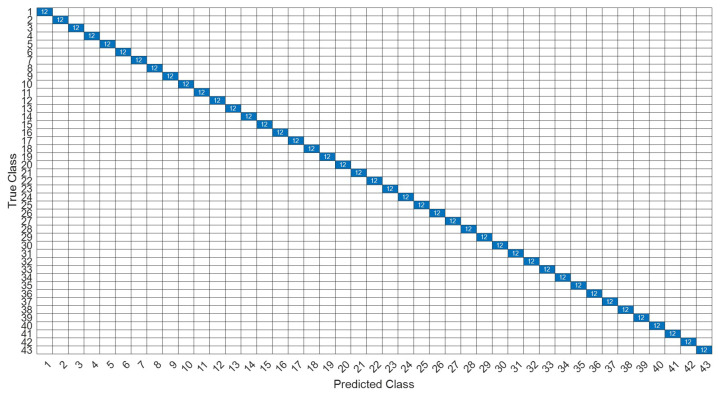
The confusion matrix of the DNN model with wavelet 40-features dataset.

**Figure 32:**
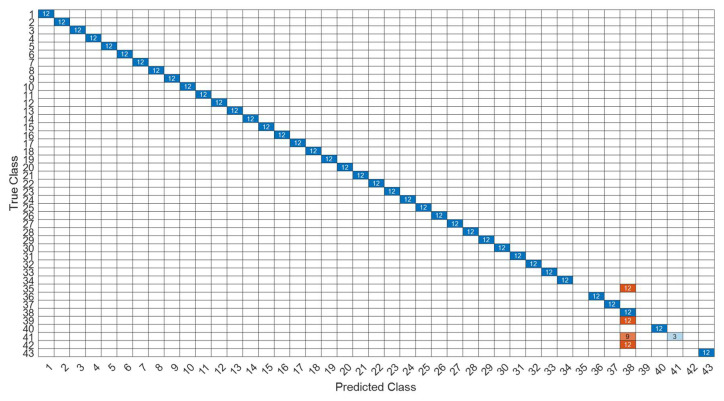
The confusion matrix of the CNN model with wavelet 40-features dataset.

**Figure 33:**
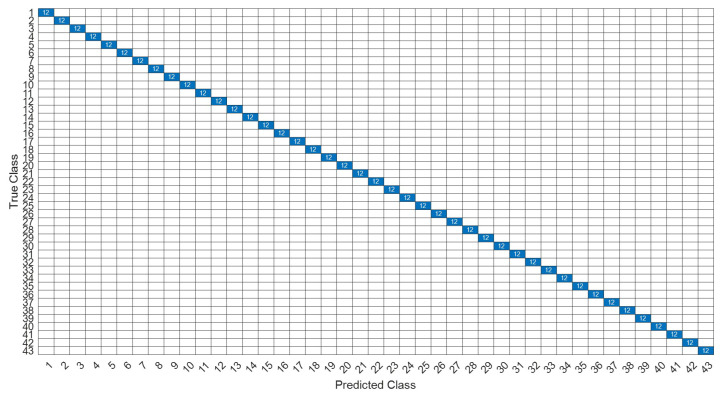
The confusion matrix of the LSTM model with wavelet 40-features dataset.

Figure 26 is a comparison of the loss functions of the LPC feature dataset with 40 features. Figure 26a–c are the loss functions using DNN, CNN and LSTM algorithms, respectively. Similar to the previous results of taking 30 feature datasets: DNN and CNN converge faster. Moreover, compared with wavelet datasets, the CNN algorithm converges more stably, whereas LSTM has extremely poor convergence here. Figure 27 shows the comparison of the loss function of 40 kinds of features by wavelet. Figure 27a–c are the loss functions using DNN, CNN and LSTM algorithms, respectively. We find that the CNN loss function converges more smoothly, and the DNN and CNN loss functions are more stable than the experimental results with 30 features. In addition, Figure 28, Figure 29 and Figure 30 are the confusion matrices of the LPC dataset with 40 features imported into DNN, CNN and LSTM algorithms, respectively, for classification and identification. Figure 31, Figure 32 and Figure 33 are the confusion matrices of the 40-features wavelet dataset imported into DNN, CNN, and LSTM algorithms, respectively, for classification and identification. The results of our experiments can also be seen from the confusion matrix. Furthermore, LSTM has faster gradient convergence than the experimental results with 30 features per failure, but is less stable when the number of iterations is small.

## 5. Conclusions

An early vehicle fault signal classification method is proposed based on voiceprint filtering combined with deep learning algorithms. We collected 43 different vehicle breakdown signals. LPC and wavelet were used to filter the original signal to obtain important signal spectral characteristics used to define fault type. In addition, three machine learning algorithms, DNN, CNN and LSTM, were used to develop automatic diagnosis methods to classify complex fault features. Looking at the whole experiment, in terms of the LPC dataset, CNN has the best performance, followed by DNN, and finally LSTM. However, in terms of model training time, the order of the three is reversed.

The LPC dataset and the CNN algorithm can obtain the best identification results, but the training process is also the most time-consuming. With regard to LPC + LSTM, although the training time is the shortest, it is almost impossible to identify and classify. That is, the accuracy rate is extremely low. Furthermore, for the wavelet dataset, DNN has the best performance both in terms of identification performance and training time. For datasets with large dimensions, the accuracy of the wavelet algorithm combined with LSTM also has good identification performance. Based on our experimental results, we can infer that in this experiment, the wavelet algorithm combined with DNN can not only achieve the best identification performance, but also the shortest model training time when the dataset dimension is large.

All deep learning models are implemented on the Python Pytorch platform using NVIDIA GeForce GTX. In this research, early failure prediction in vehicles is of great significance to the emerging Internet of Vehicles and can help increase the production capacity of Internet of Things and Industry 4.0 applications. Future work will seek to combine two filtering methods, such as MFCC + LPC or MFCC + wavelet, and to apply machine learning methods suitable for natural language processing (NLP), such as long short-term memory (LSTM) work to produce an application that effectively achieves faster identification. Voice recognition methods are an area worthy of attention and have extensive applications in daily life; thus, combining artificial intelligence and voiceprint recognition can potentially produce significant and widespread benefits.

## Figures and Tables

**Table 1:** The definition of 43 driving fault signals (engine, tire, and chassis).

Item	Feature Signal Condition	Statement
1	Tire V30 32 psi	Normal pressure in 30 km/h.
2	Tire V30 50 psi	High pressure in 30 km/h.
3	Tire V30 20 psi	Low pressure in 30 km/h.
4	Tire V30 32 psi fail	Tire wear and normal pressure in 30 km/h.
5	Tire V30 50 psi fail	Tire wear and high pressure in 30 km/h.
6	Tire V30 20 psi fail	Tire wear and low pressure in 30 km/h.
7	Tire V20 32 psi	Normal pressure in 20 km/h.
8	Tire V20 50 psi	High pressure in 20 km/h.
9	Tire V20 20 psi	Low pressure in 20 km/h.
10	Tire V20 32 psi fail	Tire wear and normal pressure in 20 km/h.
11	Tire V20 50 psi fail	Tire wear and high pressure in 20 km/h.
12	Tire V20 20 psi fail	Tire wear and low pressure in 20 km/h.
13	Tap V30 32 psi	Tire studs and normal pressure in 30 km/h.
14	Tap V30 50 psi	Tire studs and high pressure in 30 km/h.
15	Tap V30 20 psi	Tire studs and low pressure in 30 km/h.
16	Tap V20 32 psi	Tire studs and normal pressure in 20 km/h.
17	Tap V20 50 psi	Tire studs and high pressure in 20 km/h.
18	Tap V20 20 psi	Tire studs and low pressure in 20 km/h.
19	Belt 1000 rpm normal	Belt normal at 1000 rpm.
20	Belt 1500 rpm normal	Belt normal at 1500 rpm.
21	Belt 2000 rpm normal	Belt normal at 2000 rpm.
22	Belt 1000 rpm idle speed	Belt idle speed at 1000 rpm.
23	Belt 1500 rpm idle speed	Belt idle speed at 1500 rpm.
24	Belt 2000 rpm idle speed	Belt idle speed at 2000 rpm.
25	Toe-in V30 32 psi	Chassis toe-in and normal pressure in 30 km/h.
26	Toe-in V30 50 psi	Chassis toe-in and high pressure in 30 km/h.
27	Toe-in V30 20 psi	Chassis toe-in and low pressure in 30 km/h.
28	Toe-in V20 32 psi	Chassis toe-in and normal pressure in 20 km/h.
29	Toe-in V20 50 psi	Chassis toe-in and high pressure in 20 km/h.
30	Toe-in V20 20 psi	Chassis toe-in and low pressure in 20 km/h.
31	Toe-out V30 32 psi	Chassis toe-out and normal pressure in 30 km/h.
32	Toe-out V30 50 psi	Chassis toe-out and high pressure in 30 km/h.
33	Toe-out V30 20 psi	Chassis toe-out and low pressure in 30 km/h.
34	Toe-out V20 32 psi	Chassis toe-out and normal pressure in 20 km/h.
35	Toe-out V20 50 psi	Chassis toe-out and high pressure in 20 km/h.
36	Toe-out V20 20 psi	Chassis toe-out and low pressure in 20 km/h.
37	Drive V10 32 psi	Broken drive shaft boot and normal pressure in 10 km/h.
38	Drive V10 50 psi	Broken drive shaft boot and high pressure in 10 km/h.
39	Drive V10 20 psi	Broken drive shaft boot and low pressure in 10 km/h.
40	Drive V20 50 psi	Broken drive shaft boot and high pressure in 20 km/h.
41	No.1 nozzle 1000 rpm	Engine single cylinder misfire, idle speed at 1000 rpm.
42	No.1 nozzle 1500 rpm	Engine single cylinder misfire, idle speed at 1500 rpm.
43	No.1 nozzle 2000 rpm	Engine single cylinder misfire, idle speed at 2000 rpm.

**Table 2:** Results with three deep learning structures of 30-feature datasets.

Results of 30-Feature Datasets	DNN	CNN	LSTM
Accuracy for LPC	0.579	1.000	0.023
Training time for LPC (s)	97.515	115.200	70.031
Accuracy for Wavelet	1.000	0.858	0.879
Training time for Wavelet (s)	97.747	118.767	70.042

**Table 3:** Results with three deep learning structures of 40-feature datasets.

Results of 40-Feature Datasets	DNN	CNN	LSTM
Accuracy for LPC	0.828	1.000	0.023
Training time for LPC (s)	125.193	207.647	163.552
Accuracy for Wavelet	1.000	0.913	1.000
Training time for Wavelet (s)	124.090	207.727	162.962

## Data Availability

Not applicable.

## References

[1] Jaynes C., Seales W.B., Calvert K., Fei Z., Griffioen J. (2003). The Metaverse: A networked collection of inexpensive, self-configuring, immersive environments. Proceedings of the Workshop on Virtual Environments 2003 (EGVE’03).

[2] Strutynska I., Dmytrotsa L., Kozbur H., Hlado O., Dudkin P., Dudkina O. Development of Digital Platform to Identify and Monitor the Digital Business Transformation Index. Proceedings of the 2020 IEEE 15th International Conference on Computer Sciences and Information Technologies (CSIT).

[3] Jiang Y. An Analysis of the Relationship between Mechanical and Electronic Engineering and Artificial Intelligence. Proceedings of the 2019 International Conference on Virtual Reality and Intelligent Systems (ICVRIS).

[4] Ghosh A., Chakraborty D., Law A. (2018). Artificial intelligence in internet of things. CAAI Trans. Intell. Technol..

[5] Buyya R. Cloud computing: The next revolution in information technology. Proceedings of the 2010 First International Conference on Parallel, Distributed and Grid Computing (PDGC 2010).

[6] Dai X., Gao Z. (2013). From model signal to knowledge: A data-driven perspective of fault detection and diagnosis. IEEE Trans. Ind. Inform..

[7] Purkait P., Chakravorti S. (2002). Time and frequency domain analyses based expert system for impulse fault diagnosis in transformers. IEEE Trans. Dielectr. Electr. Insul..

[8] Yang F., Wang S., Li J., Liu Z., Sun Q. (2014). An overview of Internet of Vehicles. China Commun..

[9] Cummings M.L., Bauchwitz B. (2022). Safety Implications of Variability in Autonomous Driving Assist Alerting. IEEE Trans. Intell. Transp. Syst..

[10] Zhang H., Zhang Q., Liu J., Guo H. (2018). Fault detection and repairing for intelligent connected vehicles based on dynamic bayesian network model. IEEE Internet Things J..

[11] Lu Y., Huang X., Zhang K., Maharjan S., Zhang Y. (2020). Blockchain empowered asynchronous federated learning for secure data sharing in internet of vehicles. IEEE Trans. Veh. Technol..

[12] Liu H., Ma J., Xu T., Yan W., Ma L., Zhang X. (2020). Vehicle detection and classification using distributed fiber optic acoustic sensing. IEEE Trans. Veh. Technol..

[13] Obeid N.H., Battiston A., Boileau T., Nahid-Mobarakeh B. (2017). Early intermittent interturn fault detection and localization for a permanent magnet synchronous motor of electrical vehicles using wavelet transform. IEEE Trans. Transp. Electrif..

[14] Kemalkar A.K., Bairagi V.K. Engine fault diagnosis using sound analysis. Proceedings of the International Conference on Automatic Control and Dynamic Optimization Techniques (ICACDOT).

[15] Chao K.W., Chen Y.H., Ho Y.Y., Guu D.Y., Luo L.B., Tseng C.W., Su C.S., Gong C.A., Huang Q.Y., Lee I.E. Feature-Driven Fault Classification for Vehicle Driving Health based on Supervised Learning. Proceedings of the 26th National Conference on Vehicle Engineering.

[16] Gong C.S.A., Lee H.C., Chuang Y.C., Li T.H., Su C.H.S., Huang L.H., Hsu C.W., Hwang Y.S., Lee J.D., Chang C.H. (2018). Design and Implementation of Acoustic Sensing System for Online Early Fault Detection in Industrial Fans. Hindawi J. Sens..

[17] Gong C.S.A., Su C.H.S., Tseng K.H. (2020). Implementation of machine learning for fault classification on vehicle power transmission system. IEEE Sens. J..

[18] Gong C.S.A., Su C.-H.S., Chao K.-W., Chao Y.-C., Su C.-K., Chiu W.-H. (2021). Exploiting deep neural network and long short-term memory methodologies in bioacoustic classification of LPC-based features. PLoS ONE.

[19] Ameid T., Menacer A., Talhaoui H., Harzelli I. (2017). Broken rotor bar fault diagnosis using fast Fourier transform applied to field-oriented control induction machine: Simulation and experimental study. Int. J. Adv. Manuf. Technol..

[20] Ayhan T., Dehaene W., Verhelst M. A 128:2048/1536 point FFT hardware implementation with output pruning. Proceedings of the 2014 22nd European Signal Processing Conference (EUSIPCO).

[21] Yan R., Gao R.X., Chen X. (2014). Wavelets for fault diagnosis of rotary machines: A review with applications. Signal Process..

[22] Swedia E.R., Mutiara A.B., Subali M., Ernastuti Deep Learning Long-Short Term Memory (LSTM) for Indonesian Speech Digit Recognition using LPC and MFCC Feature. In Proceedings of the 2018 Third International Conference on Informatics and Computing (ICIC).

[23] Chong U.P., Lee S.S., Sohn C.H. Fault diagnosis of the machines in power plant using LPC. Proceedings of the 8th Russian-Korean International Symposium on Science and Technology (KORUS).

[24] Chao K.W., Hu N.-Z., Chao Y.-C., Su C.-K., Chiu W.-H. (2019). Implementation of artificial intelligence for classification of frogs in bioacoustics. Symmetry.

[25] Yin S., Huang Z. (2014). Performance monitoring for vehicle suspension system via fuzzy positivistic C-means clustering based on accelerometer measurements. IEEE/ASME Trans. Mechatron..

[26] Tax D.M.J., Ypma A., Duin R.P.W., Hand D.J., Kok J.N., Berthold M.R. (1999). Pump failure determination using support vector data description. Advances in Intelligent Data Analysis. IDA.

[27] Shifat T.A., Hur J.-W. (2021). ANN Assisted Multi Sensor Information Fusion for BLDC Motor Fault Diagnosis. IEEE Access.

[28] Weatherspoon M.H., Langoni D. (2008). Accurate and efficient modeling of FET cold noise sources using ANNs. IEEE Trans. Instrum. Meas..

[29] Zhang Y., Fu Y., Jiang W., Li C., You H., Li M., Chandra V., Lin Y. DIAN: Differentiable Accelerator-Network Co-Search Towards Maximal DNN Efficiency. Proceedings of the 2021 IEEE/ACM International Symposium on Low Power Electronics and Design (ISLPED).

[30] Krizhevsky A., Sutskever I., Hinton G.E. Imagenet classification with deep convolutional neural networks. Proceedings of the 26th Annual Conference on Neural Information Processing Systems.

[31] Lei X., Pan H., Huang X. (2019). A Dilated CNN Model for Image Classification. IEEE Access.

[32] Markel J.D., Gray A.H. (1976). Linear Prediction of Speech.

[33] Goswami J.C., Chan A.K. (2008). Fundamentals of Wavelets: Theory, Algorithms, and Applications.

[34] Choi J.Y., Choi C.H. (1992). Sensitivity analysis of multilayer perceptron with differentiable activation functions. IEEE Trans. Neural Netw..

[35] Fukushima K. (1980). Neocognitron: A self-organizing neural network model for a mechanism of pattern recognition unaffected by shift in position. Biol. Cybern..

[36] Olshausen B.A., Field D.J. (1996). Emergence of simple-cell receptive field properties by learning a sparse code for natural images. Nature.

[37] Szegedy C., Liu W., Jia Y., Sermanet P., Reed S., Anguelov D., Erhan D., Vanhoucke V., Rabinovich A. Going deeper with convolutions. Proceedings of the 2015 IEEE Conference on Computer Vision and Pattern Recognition (CVPR).

[38] Simonyan K., Zisserman A. (2014). Very deep convolutional networks for large-scale image recognition. arXiv.

[39] LeCun Y., Bottou L., Bengio Y., Haffner P. (1998). Gradient-based learning applied to document recognition. Proc. IEEE.

[40] Wen L., Li X., Gao L., Zhang Y. (2018). A new convolutional neural network-based data-driven fault diagnosis method. IEEE Trans. Ind. Electron..

[41] Aggarwal C.C. (2018). Neural Networks and Deep Learning.

[42] Haykin S., Veen B.V. (2003). Signals and Systems.

[43] Hotho G., Villemoes L.F., Breebaart J. (2008). A Backward-Compatible Multichannel Audio Codec. IEEE Trans. Audio Speech Lang. Process..

[44] Lin C.H., Lin Y.C., Tang P.W. (2022). ADMM-ADAM: A New Inverse Imaging Framework Blending the Advantages of Convex Optimization and Deep Learning. IEEE Trans. Geosci. Remote Sens..

[45] Glorot X., Bordes A., Bengio Y. Deep sparse rectifier neural networks. Proceedings of the 14th International Conference on Artificial Intelligence and Statistics (AISTATS).

[46] Nguyen A., Pham K., Ngo D., Ngo T., Pham L. An Analysis of State-of-the-art Activation Functions For Supervised Deep Neural Network. Proceedings of the 2021 International Conference on System Science and Engineering (ICSSE).

